# Bilateral Facial Palsy: An Atypical Neurological Complication of Varicella-Zoster Virus Reactivation

**DOI:** 10.7759/cureus.81780

**Published:** 2025-04-06

**Authors:** Reyaz Ahmad, Rohit Anand, Sushant Shangari, Reetu Singh

**Affiliations:** 1 Neurology, Tata Main Hospital, Jamshedpur, IND

**Keywords:** bilateral facial palsy, chickenpox, cranial neuropathies, varicella zoster virus reactivation, vzv

## Abstract

Bilateral facial palsy is an exceptionally rare condition, often indicative of systemic involvement or severe underlying pathology. This report details a case of bilateral facial nerve paralysis in a young adult following recent varicella infection, an association infrequently documented in clinical practice. The patient presented with classic signs of bilateral facial nerve dysfunction alongside a history of recent chickenpox infection. Diagnostic workup confirmed bilateral axonal neuropathy, suggesting a peripheral nerve etiology linked to varicella-zoster virus reactivation. This case highlights the need for vigilance in recognizing rare neurological manifestations of common infections to ensure timely diagnosis and appropriate management.

## Introduction

Facial palsy is frequently encountered in clinical practice, with unilateral involvement being most common and often attributed to idiopathic Bell's palsy, affecting 20-25 per 100,000 annually [1]. Bilateral facial palsy (BFP), however, is rare, accounting for less than 2% of facial palsy presentations, with an incidence of one in five million [2,3]. The etiological spectrum of BFP includes infectious, autoimmune, neoplastic, vascular, and metabolic causes. While Guillain-Barré syndrome and Lyme disease are well-known culprits, varicella-zoster virus (VZV) infection is an uncommon cause, particularly in adults [4]. VZV can manifest neurologically as Ramsay Hunt syndrome, but bilateral facial neuropathy secondary to VZV is exceedingly rare and sparsely reported in the literature [5]. This report discusses a case of BFP secondary to VZV reactivation, emphasizing its diagnostic and therapeutic complexities.

## Case presentation

A 34-year-old man presented to the emergency department with complaints of decreased taste sensation, deviation of the mouth toward the right side, incomplete closure of the left eye, and drooping of the mouth. The symptoms developed acutely. His history was notable for chickenpox 10 days prior, and he denied prior episodes of facial weakness, seizures, or other neurological symptoms. He had no comorbidities and denied alcohol, drug, or tobacco use.

**Figure 1:**
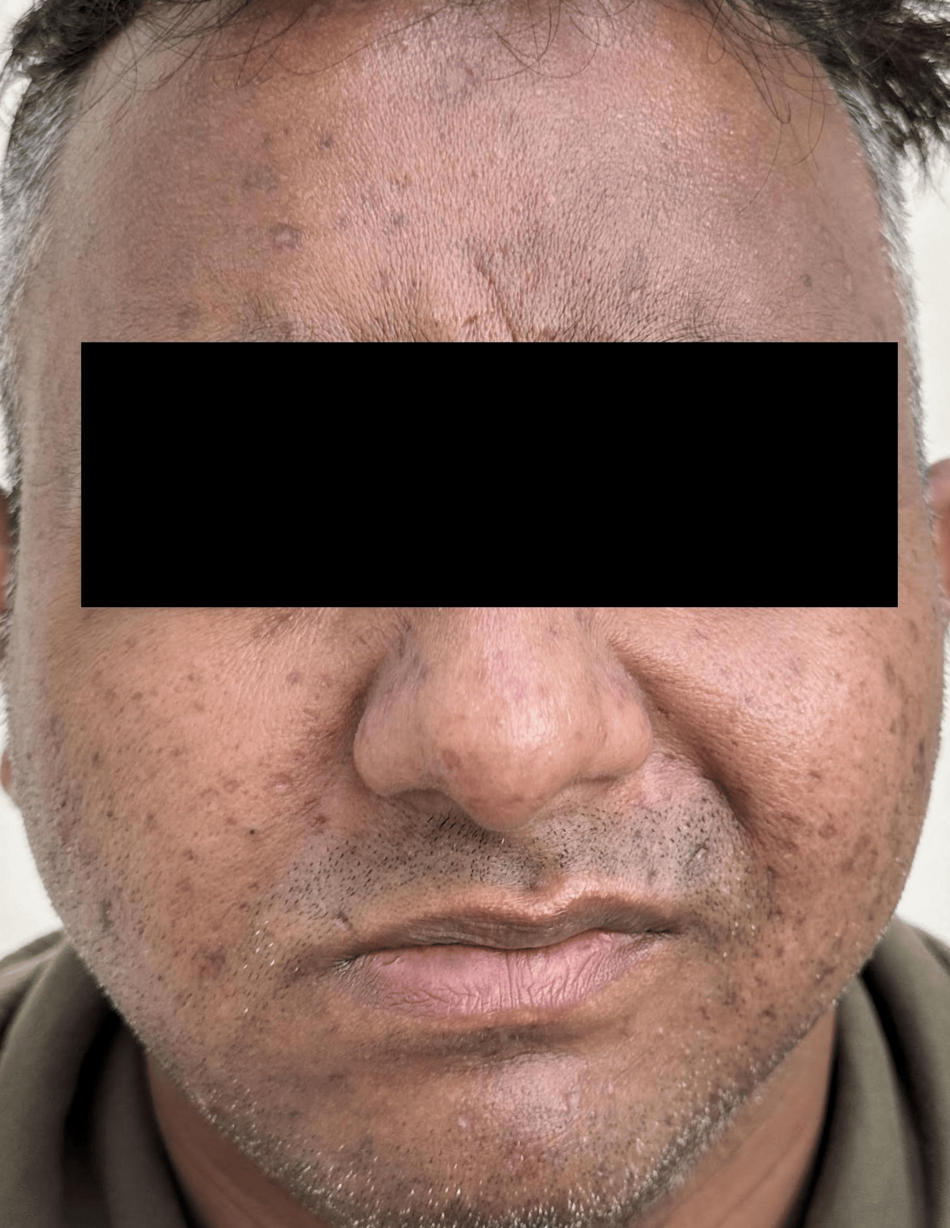
Image showing the absence of forehead wrinkling, loss of prominent nasolabial folds, and presence of healed vesicular eruptions consistent with recent chicken pox infection.

Physical examination revealed bilateral facial weakness. The patient was unable to elevate his eyebrows, puff his cheeks, smile symmetrically, or close his eyes completely (Figure 1). Neurological examination otherwise revealed no abnormalities. Laboratory investigations included a serological panel for VZV antibodies, which showed elevated IgG (3481.00 mIU/mL; reference <150 mIU/mL) and IgM (1.96 Index; reference <1.00), confirming recent VZV infection (Table 1). MRI brain showed bilateral facial nerves of normal thickness with discontinuous enhancement, a normal finding.

**Table 1:** Serological panel for VZV antibodies. CLIA: Clinical Laboratory Improvement Amendments

Test Name	Results	Units	Biological Reference Interval
Varicella Zoster Virus (VZV) Antibodies Panel (CLIA)			
VZV IgG	3481.00	mIU/mL	<150.00
VZV IgM	1.96	Index	<1.00

Additionally, the patient reported fever and multiple episodes of loose stools over the preceding 48 hours. He was diagnosed with non-infective gastroenteritis and colitis, unspecified, as part of his clinical presentation.

Electrodiagnostic studies were performed to evaluate facial nerve function. The nerve conduction study (NCS) revealed bilateral reduction in compound muscle action potential (CMAP) amplitudes in the orbicularis oris muscles, consistent with an axonal type of facial neuropathy (Table 2). However, nerve latencies were normal, excluding conduction block.

**Table 2:** Key NCS findings. R: Right, L: Left, ms: milliseconds, mV: millivolts, NCS: nerve conduction study Normal range of latency ≤4.2(ms) Normal range of amplitude ≥1(mV)

Nerve/Sites	Muscle	Latency (ms)	Amplitude (mV)
R Facial- Orbicularis oris			
Postauricular	Orbicularis oris	3.40	0.9
L Facial- Orbicularis oris			
Postauricular	Orbicularis oris	2.94	0.8

The patient was managed with antiviral therapy tablet acyclovir 800mg five times a day for seven days. Prednisolone at a dose of 1mg/kg/day was given and tapered over the next 20 days. With treatment and supportive facial physiotherapy and eye care, the patient demonstrated gradual improvement of facial weakness; however, the recovery was incomplete with residual facial asymmetry at three-month follow-up. 

## Discussion

BFP is a diagnostic challenge due to its rarity and diverse etiologies. Common causes include autoimmune diseases, infections, neoplasms, and metabolic disorders [6,7]. VZV, a neurotropic virus latent in sensory ganglia, can reactivate under immunosuppression or stress, leading to cranial neuropathies [8]. Although unilateral VZV-related facial palsy is well-documented, bilateral involvement is exceedingly rare.

VZV reactivation involves viral replication in dorsal root or cranial nerve ganglia, leading to inflammation and axonal damage. Bilateral involvement, however, is extremely rare and likely represents dissemination of the virus or simultaneous reactivation in multiple cranial ganglia, as observed in this patient.

The patient’s presentation with bilateral axonal neuropathy without other neurological deficits, coupled with serological evidence of VZV infection, supports the diagnosis of VZV-associated BFP. The pathophysiological mechanism may involve inflammation-induced axonal damage, as evidenced by reduced CMAP amplitudes on the NCS. The concurrent gastrointestinal symptoms, though atypical, may suggest systemic inflammation or viral dissemination. Previous literature has reported rare cases of varicella-zoster involvement in gastrointestinal neuropathy, though this remains speculative in our patient.

Early diagnosis relies on clinical suspicion, supported by serological markers and neurophysiological studies. Elevated VZV-specific antibodies and reduced CMAP amplitudes on NCS are diagnostic hallmarks [8,9]. Imaging and additional testing exclude alternative diagnoses, such as Guillain-Barré syndrome and sarcoidosis [10].

The cornerstone of VZV-related facial palsy management is antiviral therapy, such as valacyclovir, combined with corticosteroids to reduce inflammation [11]. Supportive measures, including artificial tears and physical therapy, aid recovery and prevent complications like corneal ulceration [12].

## Conclusions

This case highlights the importance of considering infectious causes, particularly VZV reactivation, in BFP. Prompt recognition and targeted therapy facilitated a favorable outcome in this patient. Multidisciplinary collaboration was crucial, underscoring the need for vigilance in atypical presentations of common infections. Future studies should explore the mechanisms and treatment outcomes of bilateral neuropathy in VZV infections to refine clinical practice.
